# Risk prediction of advanced colorectal neoplasia among diabetic patients: A derivation and validation study

**DOI:** 10.1002/jgh3.13062

**Published:** 2024-05-13

**Authors:** Martin CS Wong, Eman YM Leung, Harry HX Wang, Junjie Huang

**Affiliations:** ^1^ The Jockey Club School of Public Health and Primary Care, Faculty of Medicine Chinese University of Hong Kong Hong Kong SAR China; ^2^ Centre for Health Education and Health Promotion, Faculty of Medicine Chinese University of Hong Kong Hong Kong SAR China; ^3^ The School of Public Health Peking University Beijing China; ^4^ The School of Public Health, The Chinese Academy of Medical Sciences and The Peking Union Medical Colleges Beijing China; ^5^ School of Public Health Sun Yat‐Sen University Guangzhou Guangdong China; ^6^ Usher Institute, Deanery of Molecular, Genetic and Population Health Sciences The University of Edinburgh Edinburgh UK

**Keywords:** advanced colorectal neoplasia, diabetes, epidemiology, risk factor

## Abstract

**Background and Aim:**

Colorectal cancer (CRC) is the third most common cancer in the world. This study devises and validates a clinical scoring system for risk prediction of advanced colorectal neoplasia (ACN) to guide colonoscopy evaluation among diabetic patients.

**Methods:**

We identified 55 964 diabetic patients who received colonoscopies from a large database in a Chinese population (2008–2018). We recruited a derivation cohort based on random sampling. The risk factors of CRC evaluated by univariate analysis were examined for ACN, defined as advanced adenoma, CRC, or any combination thereof using binary logistic regression analysis. We used the adjusted odds ratios (aORs) for independent risk factors to devise a risk score, ranging from 0 to 6: 0–4 “average risk” (AR) and 5–6 “high risk” (HR). The other subjects acted as an independent validation cohort.

**Results:**

The prevalence of ACN in both the derivation and validation cohorts was 2.0%. Using the scoring system constructed, 78.5% and 21.5% of patients in the validation cohort were classified as AR and HR, respectively. The prevalence of ACN in the AR and HR groups was 1.5% and 4.1%, respectively. Individuals in the HR group had a 2.78‐fold increased prevalence of ACN than the AR group. The concordance (c‐) statistics was 0.70, implying a good discriminatory capability of the risk score to stratify high‐risk individuals who should consider colonoscopy.

**Conclusion:**

The clinical risk scoring system based on age, gender, smoking, presence of hypertension, and use of aspirin is useful for ACN risk prediction among diabetic patients.

## Graphical abstract

Participant scores were determined based on risk factors: age at first colonoscopy (below 60: 0 points, 60 or above: 2 points), gender (female: 0 points, male: 1 point), smoking (non‐smoker: 0 points, current/ex‐smoker: 1 point), hypertension (no: 0 points, yes: 1 point), aspirin use (yes: 0 points, no: 1 point). Scores ranged from 0 to 6, summing points for each factor. Scores of 0‐4 indicated average risk, while 5‐6 indicated high risk.

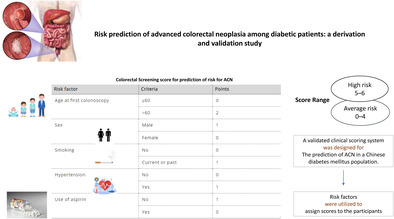

## Introduction

Colorectal cancer (CRC) is the third most commonly diagnosed cancer.^1^ In 2019, there were more than 1.9 million new CRC cases worldwide.^1^ It ranked as the second leading cause of cancer mortality, accounting for almost 1 million deaths globally in 2019.^1^ It is projected that CRC incidence and mortality will continue to increase and impose a heavy public health burden.^2^ A substantial body of evidence shows that early colonoscopy screening can ameliorate its associated morbidity and mortality by detection and removal of premalignant lesions.^3^ However, colonoscopy resources are limited and their adherence is often suboptimal when compared with other screening tools.^4^ Risk prediction scores for advanced colorectal neoplasia (ACN) could therefore prioritize high‐risk subjects for colonoscopy screening.^5^

The global prevalence of diabetes mellitus had recorded an almost fourfold increase from 108 million in 1980 to 422 million in 2014, and it was expected to rise to almost 600 million by 2035.^6^ Diabetes mellitus can lead to multisystem complications, and studies found that diabetes mellitus was associated with a higher risk of CRC.^7^ A meta‐analysis of 15 studies involving 2.5 million patients demonstrated a significantly increased relative risk (RR) (by 30%) of developing CRC in diabetes mellitus patients when compared with their healthy counterparts.^8^ Nevertheless, there is a scarcity of risk algorithms to predict ACN among diabetic patients. The proportion of the older population with diabetes mellitus was around 20%,^7^ highlighting the need for developing a risk stratification tool for this high‐risk group requiring intensive clinical attention. This study aimed to develop and validate a clinical risk stratification score for ACN prediction among diabetic patients. A simple tool for clinicians based on easy‐to‐collect information for risk stratification could assist in the identification of diabetes mellitus subjects at higher risk for ACN. It will also inform the starting age and frequency for CRC screening among diabetes mellitus patients, thereby informing the allocation of colonoscopy resources to high‐risk individuals.

## Methods

### Settings

The present study was performed in accordance with the Declaration of Helsinki and submitted to the Survey and Behavioral Research Ethics Committee of the Chinese University of Hong Kong for ethics approval. This was a population‐based retrospective cohort study with baseline recruitment between 1 January 2008 and 31 December 2018. In this study, data were extracted from Hospital Authority Data Collaboration Lab (HADCL), which is a platform providing access to an electronic healthcare database that consists of patient demographic data, clinical diagnoses, procedures, drug prescriptions, and laboratory results from all public hospitals and clinics in Hong Kong. It represents both in‐patient and out‐patient data of about 80% of the 7.49 million people in our locality. We have previously validated the database and reported a high level of completeness of patients' demographic profiles (100%) and prescription details (99.8%).^9^ The data on comorbidities were coded by the International Classification of Diseases Ninth Revision, Clinical Modification (ICD‐9‐CM) in CDARS, which have been validated to be 99% accurate concerning clinical, laboratory, imaging, and endoscopy results from the electronic medical records.^10^, ^11^, ^12^

### Study subjects

The medical condition was ascertained from the electronic healthcare database in this study. Inclusion criteria were all Chinese diabetes mellitus patients with gastrointestinal (GI) symptoms aged 18 years or older who have received at least one colonoscopy, managed in General Outpatient Clinics (GOPC) of the Hong Kong Government. Sociodemographic data including age, sex, body mass index (BMI), smoking, drinking, age at diabetes mellitus diagnosis, duration of diabetes mellitus, concomitant medical conditions, use of medications, age at first colonoscopy, histopathology findings of removed polyps, results from physical examination, relevant laboratory investigations, and drug prescriptions were collected. The duration of diabetes mellitus refers to the time since a diabetes mellitus diagnosis was made before a baseline diabetes mellitus complication screening a patient received. The age at diabetes mellitus diagnosis refers to the time difference between the self‐reported year of diabetes mellitus diagnosis during a baseline assessment and the year of birth, and the age of first colonoscopy refers to age of the patient when they received a first colonoscopy in any public hospitals. All colonoscopies in this proposed project were conducted at accredited endoscopy centers affiliated with public hospitals. The procedures were performed by experienced colonoscopy practitioners with informed consent of the screening participants. At all centers, most bowel preparation for each participant was accomplished using split‐dose polyethylene glycol. During all colonoscopy examinations, standard air insufflation was carried out. The bowel preparation regimens used at all centers were recommended by international guidelines to enhance high‐quality procedural standards. A withdrawal time of ≥6 min was targeted in each procedure to comply with the current colonoscopy quality indicators, as provided by the database. The practitioners identified and removed all lesions suitable for polypectomy, which were sent to an accredited pathology center for detailed histopathological examination. We identified patients with ACN, defined as CRC, or any colorectal adenoma with a size of ≥10 mm in diameter, high‐grade dysplasia, villous or tubulovillous histologic characteristics, or any combination of them. Patients without these codes were considered as control subjects. For subjects who received more than one colonoscopy, the first colonoscopy was considered in the analysis.

### Development of the risk scores

**Figure 1:**
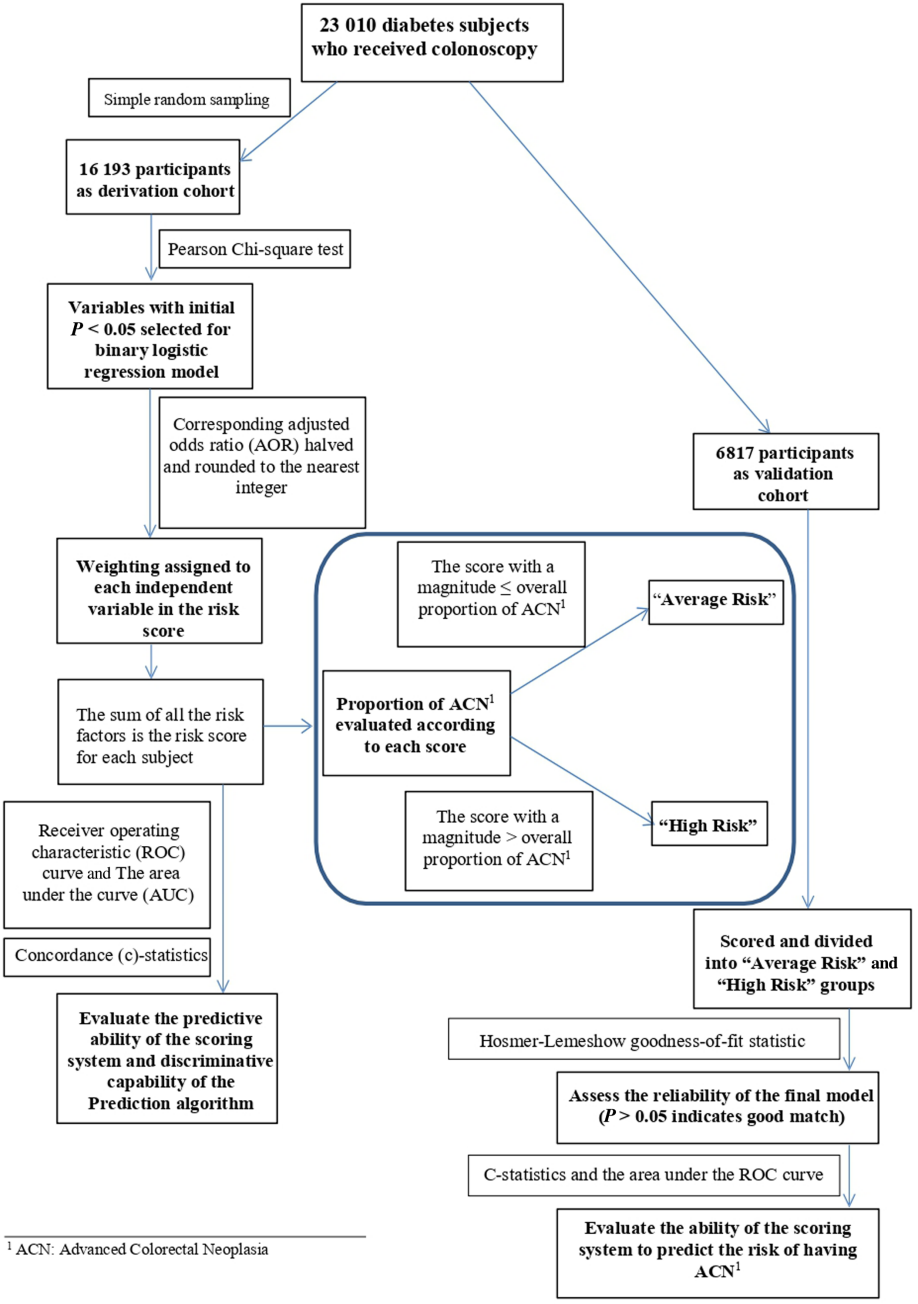
Flow diagram for development of a derivation and validation cohort.

The process of development of the risk scores is presented in Figure 1. The associations between risk factors and the colonoscopic finding of ACN were examined by Pearson chi‐square tests in the derivation cohort. Risk factors examined included age, sex, BMI, smoking (current/ex‐smoker *vs* non‐smoker), alcohol drinking (current, and social drinkers *vs* non‐drinkers), age at diabetes mellitus diagnosis, duration of diabetes mellitus, age at first colonoscopy, self‐reported medical conditions (including hypertension, ischemic heart disease, stroke, and cirrhosis), and use of medications as documented in the computerized system (including aspirin, metformin, and insulin). Variables with *P* value <0.05 in the univariate analysis were included in a binary logistic regression model, with ACN being the outcome. Each of these risk factors was then assigned a weighting in the risk score using the respective adjusted odds ratios (aOR) halved and rounded to the nearest integer to keep the simplicity. The summation of all the risk factors was the risk score for each individual. We constructed a receiver operating characteristic (ROC) curve and used the area under the curve (AUC) to examine the discriminatory capability of the score.

## Statistical analysis

Data were entered and analyzed using R version 3.5.2. The prevalence of ACN according to each score in the derivation cohort was evaluated. Scores above the overall prevalence were assigned to the “high risk” (HR) category, while those with magnitude closest to and below the overall prevalence were categorized as “average risk” (AR). Another separate binary logistic regression model was constructed using the validation cohort with the significant risk factors identified by the derivation cohort for evaluation of the aOR of each risk factor. The aORs were compared between the two cohorts. The Hosmer–Lemeshow goodness‐of‐fit test statistic was adopted for the reliability assessment of the final model, with *P* value >0.05 indicating a good match of predicted risk over observed risk. The ability of the scoring system to predict the risk of developing ACN was evaluated using the c‐statistics and the AUC of the ROC curve, with a *P* values <0.05 considered as statistically significant. To evaluate the resources required if the scoring system is implemented to refer subjects assigned as HR for polypectomy by colonoscopy, we computed the number needed to screen (NNS) to detect one ACN. It is defined as the inverse of the predicted outcome probability from the regression model.

## Results

### Participant characteristics

A total of 55 964 diabetes mellitus patients who received colonoscopy from a large database were included, consisting of 39 175 subjects in the derivation cohort and 16 789 subjects in the validation cohort (Table 1). The characteristics of the derivation cohort were similar to those of the validation cohort in terms of age at diabetes mellitus diagnosis, age at first colonoscopy, BMI, male proportion, smoking history, alcohol consumption, duration of diabetes mellitus, the prevalence of hypertension, ischemic heart disease, stroke, cirrhosis, use of aspirin, metformin, and insulin (all *P* values >0.05). The cohort of the subset refers to subjects who received a baseline diabetes mellitus complication screening between the years 2013 and 2018; the characteristics of the derivation cohort were also similar to those of the validation cohort, except for a slight difference in the prevalence of hypertension between the derivation cohort and validation cohort (66.6% *vs* 65.1% *P* = 0.037).

**Table 1:** Characteristics of participants in the derivation and validation cohorts

All patients				Subset		
	Derivation cohort *N* = 39 175	Validation cohort *N* = 16 789	*P* value	Derivation cohort *N* = 16 193	Validation cohort *N* = 6817	*P* value
Age at diabetes diagnosis (years), mean ± SD	57.92 (11.22)	57.94 (11.22)	0.793	59.33 (11.08)	59.11 (11.04)	0.173
Age at first colonoscopy (years), mean ± SD	63.91 (10.82)	63.98 (10.89)	0.458	62.05 (10.79)	61.90 (10.87)	0.352
BMI (kg/m^2^) ≥25 (%)	25.74 (4.28)	25.69 (4.10)	0.189	25.98 (4.27)	25.96 (4.25)	0.689
Sex, male, *n* (%)	20 696 (52.8)	8893 (53.0)	0.762	8779 (54.2)	3760 (55.2)	0.190
Ever smoking, *n* (%)	12 586 (32.1)	5378 (32.0)	0.826	5334 (32.9)	2285 (33.5)	0.394
Alcohol consumption^§^, *n* (%)	7830 (20.0)	3384 (20.2)	0.648	3694 (22.8)	1599 (23.5)	0.289
Duration of diabetes (years)^†^, mean ± SD	7.18 (7.54)	7.23 (7.52)	0.471	5.52 (7.30)	5.59 (7.41)	0.500
Hypertension, *n* (%)	25 983 (66.3)	10 994 (65.5)	0.054	10 777 (66.6)	4440 (65.1)	0.037
IHD/heart disease, *n* (%)	4414 (11.3)	1851 (11.0)	0.413	2095 (12.9)	867 (12.7)	0.666
Stroke, *n* (%)	2937 (7.5)	1275 (7.6)	0.688	1324 (8.2)	581 (8.5)	0.387
Cirrhosis, *n* (%)	352 (0.9)	160 (1.0)	0.535	177 (1.1)	71 (1.0)	0.730
Use of aspirin, *n* (%)	11 253 (28.7)	4781 (28.5)	0.552	5065 (31.3)	2112 (31.0)	0.656
Use of metformin, *n* (%)	29 867 (76.2)	12 884 (76.7)	0.201	11 451 (70.7)	4851 (71.2)	0.498
Use of insulin, *n* (%)	5799 (14.8)	2457 (14.6)	0.607	2308 (14.3)	995 (14.6)	0.498
Hypertension^‡^ use of aspirin, *n* (%)	8302 (21.2)	3485 (20.8)	0.248	3775 (23.3)	1536 (22.5)	0.199
Colorectal neoplasia^‡^ *n* (%)	1002 (2.6)	448 (2.7)	0.450	325 (2.0)	139 (2.0)	0.875

^†^ Time between diagnosis of diabetes and first diabetes complication screening assessment.

^‡^ ACN is defined as colorectal cancer, or any colorectal adenoma which has a size of ≥10 mm in diameter, high‐grade dysplasia, villous or tubulovillous histologic characteristics, or any combination thereof.

^§^ Alcohol drinking included current drinker, and social drinker.

BMI, body mass index; IHD, ischemic heart disease; *N*, number.

The prevalence of ACN in the derivation cohort according to risk factors is shown in Table 2. The overall prevalence of ACN was 2.0% (*n* = 325), with a higher rate in subjects who were males (2.4%), older at diabetes mellitus diagnosis (>70 years old: 3.2%), older at first colonoscopy (>60 years old: 3.1%), current or past smokers (2.6%), and hypertensive (2.3%).

**Table 2:** Distribution of risk factors among subjects with ACN and without ACN in the derivation cohort

Predictors	With ACN^§^ *N* = 325, *n* (%)	Without ACN^§^ *N* = 15 868, *n* (%)	*P* value
Sex			<0.001
Female	112 (34.5)	7302 (46.0)	
Male	213 (65.5)	8566 (54.0)	
Age at diabetes diagnosis			<0.001
18 to 49	24 (7.4)	2824 (17.8)	
50 to 55	44 (13.5)	2905 (18.3)	
56 to 60	55 (16.9)	2914 (18.4)	
61 to 65	64 (19.7)	2749 (17.3)	
66 to 70	57 (17.5)	2020 (12.7)	
>70	81 (24.9)	2456 (15.5)	
Age at first colonoscopy			<0.001
≤60	49 (15.1)	7162 (45.1)	
>60	276 (84.9)	8706 (54.9)	
BMI			0.229
<25	159 (48.9)	7230 (45.6)	
≥25 (obesity)	166 (51.1)	8638 (54.4)	
Smoking			<0.001
Never	184 (56.6)	10 675 (67.3)	
Current or past	141 (43.4)	5193 (32.7)	
Alcohol consumption^†^			0.775
No	253 (77.8)	12 246 (77.2)	
Yes	72 (22.2)	3622 (22.8)	
Duration of diabetes^‡^			0.993
<10 years	250 (76.9)	12 203 (76.9)	
≥10 years	75 (23.1)	3665 (23.1)	
Hypertension			<0.001
No	77 (23.7)	5339 (33.6)	
Yes	248 (76.3)	10 529 (66.4)	
IHD/heart disease			0.155
No	292 (89.8)	13 806 (87.0)	
Yes	33 (10.2)	2062 (13.0)	
Stroke			0.261
No	293 (90.2)	14 576 (91.9)	
Yes	32 (9.8)	1292 (8.1)	
Use of aspirin			0.494
No	229 (70.5)	10 899 (68.7)	
Yes	96 (29.5)	4969 (31.3)	
Use of metformin			0.919
No	96 (29.5)	4646 (29.3)	
Yes	229 (70.5)	11 222 (70.7)	
Use of insulin			0.070
No	290 (89.2)	13 595 (85.7)	
Yes	35 (10.8)	2273 (14.3)	

^†^ Alcohol drinking included current drinker, and social drinker.

^‡^ Time between diagnosis of diabetes and first diabetes complication screening assessment.

^§^ ACN is defined as colorectal cancer, or any colorectal adenoma which has a size of ≥10 mm in diameter, high‐grade dysplasia, villous or tubulovillous histologic characteristics, or any combination thereof.

BMI, body mass index; IHD, ischemic heart disease.

### Independent predictors of ACN in the derivation cohort

The results of univariate and multivariate binary logistic regression analysis are shown in Table 3. In the univariate analysis, age at diabetes mellitus diagnosis from 50 years old (crude odds ratio [COR] 3.34–7.27), age at first colonoscopy from 60 years onward (COR 3.91, 95% confidence interval [CI] 3.09–4.96), male gender (COR 1.62, 95% CI 1.29–2.04), smoking (COR 1.58, 95% CI 1.26–1.97), and hypertension (COR 1.63, 95% CI 1.26–2.11) were significantly associated with the detection of ACN.

**Table 3:** Univariate and multivariate predictors of ACN in the derivation cohort

		*N*_0_	*N*_1_	*N*_2_	*N*_3_ (Final)	*N*_4_
Factors	Unadjusted OR (95% CI)	aOR (95% CI)	aOR (95% CI)	aOR (95% CI)	aOR (95% CI)	aOR (95% CI)
Age at diabetes diagnosis, years						
18–39	1	1	‐	‐	‐	‐
40–49	2.24 (0.67–7.50)	1.96 (0.58–6.59)	**‐**	**‐**	**‐**	**‐**
50–55	**3.34 (1.03–10.77)**	2.73 (0.84–8.84)	**‐**	**‐**	**‐**	**‐**
56–60	**4.16 (1.30–13.31)**	2.61 (0.81–8.42)	**‐**	**‐**	**‐**	**‐**
61–65	**5.05 (1.58–16.10)**	2.26 (0.70–7.31)	**‐**	**‐**	**‐**	**‐**
66–70	**6.22 (1.94–19.89)**	2.37 (0.73–7.69)	**‐**	**‐**	**‐**	**‐**
>70	**7.27 (2.29–23.05)**	2.57 (0.80–8.29)	**‐**	**‐**	**‐**	**‐**
Age at first colonoscopy >60, years	**3.91 (3.09–4.96)**	**4.23 (3.00–5.96)**	**4.41 (3.25–6.01)**	**4.67 (3.43–6.36)**	**4.64 (3.41–6.32)**	**4.61 (3.39–6.29)**
BMI ≥25 (Obesity)	0.87 (0.70–1.09)	‐	‐	‐	‐	‐
Sex, male	**1.62 (1.29–2.04)**	**1.43 (1.09–1.88)**	**1.43 (1.09–1.88)**	**1.42 (1.08–1.86)**	**1.45 (1.10–1.91)**	**1.45 (1.10–1.91)**
Smokers/ex‐smokers	**1.58 (1.26–1.97)**	1.29 (0.99–1.67)	1.29 (0.99–1.67)	**1.32 (1.01–1.72)**	**1.31 (1.01–1.70)**	**1.31 (1.01–1.71)**
Alcohol drinking^§^	0.96 (0.74–1.25)	‐	**‐**	**‐**	‐	**‐**
Duration of diabetes^†^, years	0.99 (0.98–1.01)	‐	**‐**	**‐**	‐	**‐**
Hypertension	**1.63 (1.26–2.11)**	**1.32 (1.02–1.72)**	**1.34 (1.03–1.74)**	**1.35 (1.04–1.76)**	**1.38 (1.06–1.79)**	**1.49 (1.09–2.02)**
IHD/heart disease	0.76 (0.53–1.09)	**‐**	**‐**	‐	**‐**	**‐**
Stroke	1.23 (0.85–1.78)	**‐**	**‐**	**‐**	**‐**	**‐**
Cirrhosis	0.56 (0.14–2.24)	**‐**	**‐**	**‐**	**‐**	**‐**
Use of aspirin	0.92 (0.72–1.17)	**‐**	**‐**	**0.72 (0.56–0.92)**	**0.69 (0.54–0.88)**	0.86 (0.52–1.43)
Use of metformin	0.99 (0.78–1.26)	**‐**	**‐**	‐	**‐**	**‐**
Use of insulin	0.72 (0.51–1.03)	**‐**	**‐**	0.71 (0.49–1.01)	**‐**	**‐**
Hypertension*use of aspirin	**‐**	**‐**	**‐**	‐	**‐**	0.75 (0.42–1.34)
Model AUC		0.6723	0.6755	0.6947	0.6955	0.6889

^§^ Alcohol drinking included current drinker, and social drinker.

^†^ Time between diagnosis of diabetes and first diabetes complication screening assessment.

* ACN is defined as colorectal cancer, or any colorectal adenoma which has a size of ≥10 mm in diameter, high grade dysplasia, villous or tubulovillous histologic characteristics, or any combination thereof. Values in bold refer to the results with p values less than 0.05.

Abbreviations: aOR, adjusted odds ratio; AUC, area under the curve; BMI, body mass index; IHD, ischaemic heart disease; OR, odds ratio.

The results of the best multivariate analysis model (AUC = 0.696, Hosmer–Lemeshow goodness‐of‐fit test: *P* value = 0.4492) showed that age at first colonoscopy from 60 years onward (AOR 4.64, 95% CI 3.41–6.32), male gender (AOR 1.45, 95% CI 1.10–1.91), smoking (AOR 1.31, 95% CI 1.01–1.70), hypertension (AOR 1.38, 95% CI 1.06–1.79), and the use of aspirin (AOR 0.69, 95% CI 0.54–0.88) were significantly associated with the detection of ACN. Moreover, the results of the c‐statistics (AUC) were at 0.70.

### Development of the risk score

From the AORs of the derivation cohort, the following risk factors were utilized to assign scores to the participants (Table 4): age at first colonoscopy below 60 years old (0), 60 years old or above (2); female gender (0), male gender (1); non‐smoker (0), current/ex‐smoker (1); no hypertension (0), hypertension (1); and use of aspirin (0), no use of aspirin (1). The score of each participant ranges from 0 to 6, and the total score of each participant was the sum of all points allocated to each risk factor.

**Table 4:** Colorectal Screening score for prediction of risk for ACN^†^

Risk factor	Criteria	Points
Age at first colonoscopy	≤60	0
	>60	2
Sex	Male	1
	Female	0
Smoking	No	0
	Current or past	1
Hypertension	No	0
	Yes	1
Use of aspirin	No	1
	Yes	0

^†^ ACN is defined as colorectal cancer, or any colorectal adenoma which has a size of ≥10 mm in diameter, high‐grade dysplasia, villous or tubulovillous histologic characteristics, or any combination thereof.

The proportion of subjects having different scores is shown in Table 5. The proportion of participants with scores 0–6 was 1.0%, 10.3%, 18.0%, 24.0%, 24.8%, 15.2%, and 6.6%, respectively. The prevalence of ACN in participants with a score of 0–6 was 0.0%, 0.4%, 0.7%, 1.4%, 2.3%, 4.0%, and 4.7%, respectively. The prevalence of ACN of score 4 is the closest to the overall prevalence in the derivation cohort (2.3% *vs* 2.0%). Therefore, scores of 0–4 were classified as “AR,” while scores of 5–6 were designated as “HR” because they had a higher prevalence of ACN than that of all study participants. Using this stratification method, 78.2% of patients in the derivation cohort had average risk, and the remaining 21.8% of patients had high risk.

**Table 5:** Distribution for each score category in the derivation cohort

Score	Total No. of subjects, *n*	No. of subjects with ACN^†^, *n*	Prevalence of ACN^†^, %	
0	162	0	0.0	AR
1	1674	7	0.4	AR
2	2919	21	0.7	AR
3	3888	55	1.4	AR
4	4020	92	2.3	AR
5	2454	99	4.0	HR
6	1076	51	4.7	HR

^†^ ACN is defined as colorectal cancer, or any colorectal adenoma which has a size of ≥10 mm in diameter, high‐grade dysplasia, villous or tubulovillous histologic characteristics, or any combination thereof.

ACN, advanced colorectal neoplasia; AR, average‐risk; HR, high‐risk.

### Validity and reliability of the model

Out of the 325 ACN detected in the derivation cohort, 175 were categories into AR and 150 into HR (Table 6). The prevalence of ACN was higher in the HR group (4.2%) than in the AR group (1.4%). From the validation cohort, 78.5% and 21.5% were in the AR and HR category, respectively, with proportions similar to the subjects in the derivation cohort. For the 139 cases in the validation cohort, 79 and 60 patients were categorized as AR and HR, respectively. The prevalence of ACN in the AR and HR tiers was 1.5% (95% CI 1.15–1.80%) and 4.1% (95% CI 3.08–5.12%), respectively. Compared with participants in the AR group, subjects in the HR group had a statistically significantly higher risk of ACN (RR 2.78, 95% CI 2.00–3.86%, *P* = 0.005). The NNS was 24 and 67 for HR and AR groups, respectively.

**Table 6:** Prevalence of advanced colorectal neoplasia (ACN) by risk tier

	Derivation cohort		Validation cohort		
Risk tier (risk score)	No. of subjects (%)	Colorectal neoplasia^†^ (%) (95% CI)	No. of subjects (%)	Colorectal neoplasia^†^ (%) (95% CI)	Relative risk (95% CI)
Average‐risk (score range: 0–4)	12 663 (78.2)	175 (1.4)	5354 (78.5)	79 (1.5)	1
		(1.18–1.59)		(1.15–1.80)	
High‐risk (5–6)	3530 (21.8)	150 (4.2)	1463 (21.5)	60 (4.1)	2.78 (2.00–3.86) *P* = 0.005
		(3.58–4.91)		(3.08–5.12)	
Total	16 193 (100)	325 (2.0)	6817 (100)	139 (2.0)	
		(1.79–2.22)		(1.70–2.37)	

^†^ ACN is defined as colorectal cancer, or any colorectal adenoma which has a size of ≥10 mm in diameter, high‐grade dysplasia, villous or tubulovillous histologic characteristics, or any combination thereof.

CI, confidence interval.

## Discussion

This study devised and verified ACN risk prediction using patients' age, gender, smoking habits, the presence of hypertension, and the use of aspirin as scoring criteria and evaluation items. We found that the scoring system could successfully stratify diabetes mellitus patients into average risk (1.5%) *versus* the high risk of harboring ACN (3.6%) with a good discriminatory capability (c‐statistics 0.70). We used sophisticated multivariate regression analysis to select the model (*N*_0_ − *N*_4_) with the best goodness of fit and therefore the highest reliability. When the scoring system is applied to diabetes mellitus patients, the NNS was also small for preventing adverse cases from occurring, suggesting that its implementation could lead to efficient use of colonoscopy.

A recent systematic review and meta‐analysis have been performed to summarize available evidence on risk scores for the prediction of ACN among populations undergoing colonoscopy.^13^ They have identified 22 studies consisting of 17 original risk scores. The median number of predictor variables was five, and the most commonly included predictors include age, sex, family history of CRC in first‐degree relatives, BMI, and smoking habits. The c‐statistics, or AUC in binary logistic regression analysis, ranged from 0.62 to 0.77 in individual studies and 0.61 to 0.70 in the pooled analysis. The present risk score contains five predictors with relatively high c‐statistics, allowing an accurate prediction of ACN. To our knowledge, there has been only one study that developed a risk score for the prediction of ACN for diabetes mellitus patients.^14^ The algorithm generated seven predictors, including age, gender, BMI, age of diabetes mellitus onset, the use of antidiabetic treatments, HbA1c, and C‐reactive protein. Nonetheless, that study involved a single center with a relatively modest sample size. Also, the performance of that scoring algorithm has not yet been evaluated, and no studies have presented a validated score that could be directly applied in clinical practice for diabetes mellitus patients. Otherwise, previous algorithms focused on average‐risk general populations. Development of such a risk scoring system for diabetes mellitus patients could therefore address an important knowledge gap, as diabetes mellitus has been well recognized as a significant risk factor for ACN. Previous literature showed that diabetes mellitus patients had a higher risk for CRC than their healthy counterparts in China (OR = 4.97).^15^ Endogenous hyperinsulinemia has been speculated as one possible mechanism driving the higher risk of CRC among type 2 diabetes mellitus patients.^16^ In addition, insulin could lead to cell proliferation and reduce apoptosis by enhancing the bioavailability of Insulin‐like Growth Factor‐1 (IGF‐1).^17^ Existing literature in general supports a “hyperinsulinemia‐colorectal cancer” sequence, and the evidence comes from experimental and epidemiologic data.^18^, ^19^, ^20^ High serum levels of insulin have been independently related to an increased risk of CRC.^21^, ^22^ Therefore, in addition to common risk factors, our study also considered other possible risk factors that are related to diabetes mellitus for ACN, such as age at diabetes mellitus diagnosis, duration of diabetes mellitus, and concomitant medical conditions, which focus more on patients with diabetes mellitus and hope to build a more comprehensive ACN prediction model for patients with diabetes mellitus.

This clinical risk score is easy to use by primary care practitioners including clinicians, nurse educators, allied health professionals, and patients who are considering receiving colonoscopy for early detection of ACN. The data needed to assess ACN risk in this score are user‐friendly in both clinical and community settings, as all of this elementary information can be collected by patient self‐reports. The contribution of this risk algorithm to the current literature is unique, as it could evaluate the risks of ACN for individual patients, which provides an objective basis for their decision to receive colonoscopy. For instance, diabetes mellitus patients assigned as having a high risk for ACN could choose an earlier colonoscopy, while those at average risk might choose surveillance or noninvasive tests that are less sensitive to ACN, such as fecal immunochemical tests (FITs). This prediction algorithm may improve colonoscopy yield and optimize the cost‐effectiveness of colonoscopy workup. Furthermore, identification of the precursors of CRC such as ACN carries the benefits of colonoscopy by early detection of removal lesions, thus improving patients' quality of life and survival rates.^14^ It has previously been shown that the knowledge of one's risks for CRC could influence one's screening behavior over time.^23^ Therefore, the adoption of this scoring system in clinical consultations might also enhance risk communications between patients and the attending physicians, which could potentially optimize patients' awareness of their own risk of ACN and adherence to colonoscopy workups.

## Strengthens and limitations

This study has collected patient information over a long period, and the large sample size represents one of its strengths. Individuals are assigned to derivation or validation cohorts using computer‐generated random numbers; thus, this cohort allocation process is designed to ensure unbiased representation within each cohort group, thereby minimizing the risk of selection bias. In addition, both the predictors and colonoscopy outcomes are based on objective data retrieved from the computer system, which has proven to be complete and accurate in our previous evaluations.^9^ Nevertheless, there are several limitations which should be addressed. First, although each diabetes mellitus patient recruited in this study did not have any CRC symptoms such as rectal bleeding and change of bowel motion, these complaints might not have been coded in the computer system, as they could have been written in the free text consultation notes in the computerized system. However, we have previously validated this dataset and found that the proportion of data completeness was up to 99.98%. In addition, we have not taken into account other recognized risk factors of CRC, such as dietary habits,^24^, ^25^ physical activity, sedentary behaviors, and waist circumference, which have been considered more accurate than BMI to predict CRC.^26^ Yet collection of these parameters requires validated questionnaires, which are often time‐consuming and infeasible, especially in busy clinical settings. Third, although the risk scoring system may inform the relative urgency of colonoscopy workup based on the risk of ACN, our study does not prescribe a particular period where colonoscopy should be arranged for subjects with HR and AR for ACN. Lastly, all patients in this study were ethnic Chinese, and thus the generalizability of the findings to other ethnic groups may be limited.^27^, ^28^ A multinational prospective cohort study involving subjects of nine ethnicities reported an increased risk for ACN among Japanese, Korean, and Chinese relative to other ethnic groups.^29^ As the risk of ACN in symptomatic and asymptomatic adults differs, our results were only based on the symptomatic population and hoped to develop into a comprehensive one for asymptomatic diabetic patients.

## Conclusion

In sum, we have devised a validated clinical scoring system for the prediction of ACN in a Chinese diabetes mellitus population. Its use may be associated with more efficient use of colonoscopy and contribute to early detection of ACN among high‐risk diabetes mellitus patients. External validation of the score should be performed in other population groups with different characteristics. Future studies should examine its feasibility, acceptability, cost‐effectiveness, and proportion of CRC reduction when implemented in clinical and community settings. Inclusion of other predictors such as polygenic risk score and other emerging risk factors of ACN could be performed in future evaluations.

## Ethics approval statement

This study was performed in accordance with the Declaration of Helsinki and submitted to the Survey and Behavioral Research Ethics Committee of the Chinese University of Hong Kong for ethics approval.

## Data Availability

Data are available up on reasonable request from the corresponding author.
