# Correction to “Risk Prediction of Advanced Colorectal Neoplasia Among Diabetic Patients: A Derivation and Validation Study”

**DOI:** 10.1002/jgh3.70213

**Published:** 2025-07-21

M. C. S. Wong, E. Y. M. Leung, H. H. X. Wang, and J. Huang. “Risk Prediction of Advanced Colorectal Neoplasia Among Diabetic Patients: A Derivation and Validation Study,” *JGH Open* 8, no. 5 (2024): e13062, https://doi.org/10.1002/jgh3.13062.

The authors would like to correct the authorship of their paper by adding Sarah Tsz Yui Yau, who contributed to the analysis of this project, to the author list.

This update to the author list has also been made on the article directly.

We apologize for this error.

